# Long-Term Outcomes and Prognosticators of Pediatric Primary Dilated Cardiomyopathy in an Asian Cohort

**DOI:** 10.3389/fped.2021.771283

**Published:** 2021-11-02

**Authors:** Po-Yuan Wang, Wei-Chieh Tseng, Chun-Min Fu, Mei-Hwan Wu, Jou-Kou Wang, Yih-Sharng Chen, Nai-Kuan Chou, Shoei-Shen Wang, Shuenn-Nan Chiu, Ming-Tai Lin, Chun-Wei Lu, Chun-An Chen

**Affiliations:** ^1^Department of Pediatrics, National Taiwan University Children's Hospital, Taipei, Taiwan; ^2^Department of Pediatrics, Taipei City Hospital Renai Branch, Taipei, Taiwan; ^3^Department of Pediatrics, National Taiwan University Hospital Hsin-Chu Branch, Hsin-Chu City, Taiwan; ^4^Department of Cardiovascular Surgery, National Taiwan University Hospital, Taipei, Taiwan; ^5^Department of Cardiovascular Surgery, Fu Jen Catholic University Hospital, New Taipei City, Taiwan

**Keywords:** dilated cardiomyopathy, epidemiology - analytic (risk factors), outcome, pediatric, Asian - Chinese

## Abstract

**Background:** Dilated cardiomyopathy (DCM) is the most common childhood cardiomyopathy. The epidemiological profiles and prognosticators of clinical outcomes in Asian populations are not well elucidated.

**Methods:** Data of 104 children aged <18 years with a diagnosis of primary DCM from January 1990 to December 2019 in our institutional database were retrospectively investigated. Relevant demographic, echocardiographic, and clinical variables were recorded for analysis. A *P* <0.05 was considered statistically significant.

**Results:** The median age at diagnosis was 1.4 years (interquartile range = 0.3–9.1 years), and 52.9% were males. During a median follow-up duration of 4.8 years, 48 patients (46.2%) were placed on the transplantation waitlist, and 52.1% of them eventually received heart transplants. An exceptionally high overall waitlist mortality rate was noted (27.1%), which was even higher (43.5%) if the diagnostic age was <3 years. The 1-, 5-, and 10-year transplant-free were 61.1, 48.0, and 42.8%. Age at diagnosis >3 years and severe mitral regurgitation at initial diagnosis were independent risk factors for death or transplantation (hazard ratios = 2.93 and 3.31, respectively; for both, *P* <0.001). In total, 11 patients (10.6%) experienced ventricular function recovery after a median follow-up of 2.5 (interquartile range = 1.65–5) years. Younger age at diagnosis was associated a higher probability of ventricular function recovery.

**Conclusions:** Despite donor shortage for heart transplantation and subsequently high waitlist mortality, our data from an Asian cohort indicated that transplant-free long-term survival was comparable with that noted in reports from Western populations. Although younger patients had exceptionally higher waitlist mortality, lower diagnostic age was associated with better long-term survival and higher likelihood of ventricular function recovery.

## Introduction

Dilated cardiomyopathy (DCM), characterized by left ventricular (LV) dysfunction and dilatation, is the most common childhood cardiomyopathy (1, 2). According to large-scale registry data from Australia and the United States, the incidence of newly diagnosed DCM was approximately 0.6 per 100 000 individuals per year in children younger than 18 years (1, 3). Although previous reports have indicated that LV size and function have normalized in approximately one-fourth to one-third of these patients at follow-up (4, 5), DCM remains associated with a significantly high morbidity and mortality in children (3–5). Approximately one-half of patients die or require heart transplantation within 5 years of the initial diagnosis (3, 4). Several prognosticators for recovery and death or transplantation in childhood DCM have been proposed in reports from western countries (3–5). However, little information is available regarding Asian pediatric populations (6), and detailed analyses of clinical outcomes are virtually absent. This study aimed to clarify the clinical spectrum and long-term outcomes of childhood primary DCM and to explore the potential outcome prognosticators in a large Asian patient cohort.

## Methods

### Study Population

In total, 194 patients with a DCM diagnosis (*International Classification of Diseases, Ninth Revision, Clinical Modification* codes 425.4 and *International Classification of Diseases, Tenth Revision, Clinical Modification* code I42.0), who were younger than 18 years, and who were in our institutional database from January 1990 to December 2019 served as the participants of this study. On the basis of echocardiography findings at diagnosis, only patients with both LV ejection fraction (EF) <45% and LV end-diastolic dimension (EDD) *Z* score > 2 were included. Those with secondary DCM—such as inflammatory diseases, myocardial ischemia (e.g., anomalous origin of the left coronary artery from the pulmonary artery), neuromuscular disorders, chronic cardiac pacing, and pre-excitation-related DCM—were excluded. Furthermore, those with mixed forms of DCM, including DCM with restrictive or hypertrophic features, were excluded. Finally, 104 patients with primary DCM were included in this study. The institutional review board approved this study protocol and waived the need for written informed consent.

### Clinical Data Collection

Demographic data were collected from medical records, including age at diagnosis, sex, symptoms at presentation, electrocardiography (ECG), echocardiography findings, and cardiac medication types. The QRS duration and T wave anomalies on 12-lead ECG at initial diagnosis were recorded, and QRS prolongation was defined as a value above the 95th percentile for age (7). On the basis of the echocardiography examination at the initial diagnosis, LV EDD and LV end-systolic dimension (ESD) data were collected and converted into *Z* scores (8). The LV EF was calculated using the Teichholz formula in a standard parasternal long-axis view. Mitral regurgitation (MR) was categorized into four severity groups (no, mild, moderate, and severe) using the American Society of Echocardiography guidelines (9). Moreover, the surgical and transplantation history were recorded, including surgery types, being on the waiting list, age at transplantation, and transplantation outcomes.

### Clinical Outcomes

The primary outcome of this study was a composite of all-cause mortality or heart transplantation. The secondary outcome was recovery of LV function at the follow-up, which was defined as LV EF > 45% and LV EDD *Z* score < 2 in at least two consecutive echocardiography examinations (5). Patients whose cardiac function improved after either cardiac resynchronization therapy or surgical intervention were not included.

### Statistics

Variables are summarized as the mean ± standard deviation, median and interquartile range (IQR), or numbers and percentages, as appropriate. To investigate potential changes in medical care and clinical outcomes over time, the study period was divided equally into an early era (1990–2004) and late era (2005–2019) on the basis of year of diagnosis. Comparisons between groups were performed using the independent-samples *t* test or Mann–Whitney *U* test for continuous variables and using the chi-squared or Fisher's exact test for categorical variables, as appropriate. The Kaplan–Meier analysis and log-rank test were used for survival analysis. Survival analysis with Bonferroni correction was employed for *post-hoc* comparison of survival of different risk groups (10). To compare and to estimate the cumulative incidence of LV function recovery, persistently abnormal LV function, and death/heart transplant, non-parametric competing-risks methodology was used. To explore the factors related to the primary and secondary outcomes, univariate correlates with *P* < 0.1 in the Cox proportional hazard regression analysis were selected for the multivariate regression model. We used the receiver operating characteristics curve to identify potential cutoff values for age that were predictive of clinical outcomes. Further subgroup analysis was performed through the classification of patients into younger and older groups. The analysis was conducted using SAS University Edition and SPSS (version 20 for Windows) for the statistical analyses. *P* < 0.05 was considered statistically significant.

## Results

### Patient Characteristics

The demographic characteristics of the study population (*N* = 104) and comparisons of the early and late eras are presented in Table 1. The median age at diagnosis was 1.4 years (IQR = 0.3-9.1 years), and 55 of the patients (52.9%) were males. In total, 22 patients (21.2%) had severe MR, and 19 (18.3%) had QRS prolongation at initial diagnosis. *T* wave abnormality, such as biphasic or inverted *T* waves in lateral leads, was noted in 45 patients (43.3%). The mean LV EDD and LV ESD *Z* scores were 7.13 ± 3.41 and 10.11 ± 3.91, respectively. The mean LV EF was 29.6% ± 9.3%. Patients who received a DCM diagnosis in the early era had greater LV size at the time of diagnosis. Significant differences in the prescriptions of cardiac medications were also noted between these 2 year-based groups. Digoxin was more commonly prescribed in the early era than in the late era (94.1 vs. 64.2%, *P* < 0.001), whereas beta-blockers were more often prescribed in the late era (37.3 vs. 62.3%, *P* = 0.011).

**Table 1:** Demographic characteristics of the study population and comparisons between early and late eras.

	**All** **(*n* = 104)**	**Early era** **(*n* = 51)**	**Late era** **(*n* = 53)**	***p*-values**
Age at diagnosis, median (IQR) (years)	1.4 (0.3–9.1)	1.2 (0.3–6)	1.7 (0.5–9.6)	
Male, *n* (%)	55 (52.9)	25 (49.0)	30 (56.6)	0.43
QRS prolongation, *n* (%)	19 (18.3)	12 (23.5)	7 (13.2)	0.17
T wave abnormality, *n* (%)	45 (43.3)	19 (37.3)	26 (49.1)	0.23
**Echocardiography**				
**MR Grading**				0.064
No, *n* (%)	10 (9.6)	7 (13.7)	3 (5.7)	
Mild, *n* (%)	41 (39.4)	17 (33.3)	24 (45.3)	
Moderate, *n* (%)	31 (29.8)	12 (23.5)	19 (35.8)	
Severe, *n* (%)	22 (21.2)	15 (29.4)	7 (13.2)	
LV EDD, *Z* scores	7.13 ± 3.41	7.9 ± 3.2	6.4 ± 3.5	**0.03**
LV ESD, *Z* scores	10.1 ± 3.9	11.3 ± 4.1	9.0 ± 3.5	**<0.01**
LV EF	29.6 ± 9.3	28.2 ± 9.2	30.9 ± 9.3	0.14
**Medications** Loop diuretics, *n* (%) Digoxin, *n* (%) ACEi/ARB, *n* (%) Beta-blockers, *n* (%) Spironolactone, *n* (%) Intravenous inotropes, *n* (%) ECMO, *n* (%) LVAD, *n* (%)	94 (90.4) 82 (78.8) 88 (84.6) 52 (50.0) 44 (42.3) 67 (64.4) 17 (16.3) 16 (15.4)	49 (96.1) 48 (94.1) 45 (88.2) 19 (37.3) 20 (39.2) 37 (72.5) 7 (13.7) 4 (7.8)	45 (84.9) 34 (64.2) 43 (81.1) 33 (62.3) 24 (45.3) 30 (56.6) 10 (18.9) 12 (22.6)	0.05 **<0.01** 0.28 **0.01** 0.53 0.09 0.49 0.06
Mitral valve repair, *n* (%)	11 (10.6)	4 (7.8)	7 (13.2)	0.53

*ACEi, angiotensin converting enzyme inhibitors; ARB, angiotensin receptor blockers; IQR, interquartile range; ECMO, extracorporeal membrane oxygenation; LVAD, left ventricular assist device; LV EDD, left ventricular end-diastolic dimension; LV EF, left ventricular ejection fraction; LV ESD, left ventricular end-systolic dimension; MR, mitral regurgitation*.

*P < 0.05 were shown in bold*.

During the median follow-up duration of 4.8 years (IQR = 0.5-10.4 years), 67 patients (64.4%) had history of requiring intravenous inotropes, and cardiopulmonary resuscitation was applied in 25 patients (24.0%). Mitral valve repair was attempted in 11 patients. Among 11 patients who received mitral valve repair, four patients died, four had transplant. Among the other three patients survived without transplantation, two had persistently abnormal LV function, and one experienced LV function recovery.

Extracorporeal membrane oxygenation (ECMO) was used in 17 patients (16.3%), and an LV assist device (LVAD) was implanted in 16 patients (15.4%). Among 17 ECMO uses, 11 of them was later bridged to LVAD. Among patients who received either ECMO or LVAD (n = 22), 13 patients died and six received heart transplant. The other three patients were still alive.

### Transplantation

In our cohort, 48 patients (46.2%) with advanced-stage heart failure were placed on the national transplantation waitlist. However, only 25 of them (52.1%) eventually received a heart transplant after a waiting duration of 14.9 ± 21.5 months. Thirteen patients died while on the waitlist, and six patients lost to follow-up after being listed on the waitlist. One patient had LV recovery 2 years later, and the other one received cardiac resynchronization therapy with fairly good response. The other two patients still had subnormal LV function and are still on the transplant waitlist.

### Mortality

**Figure 1:**
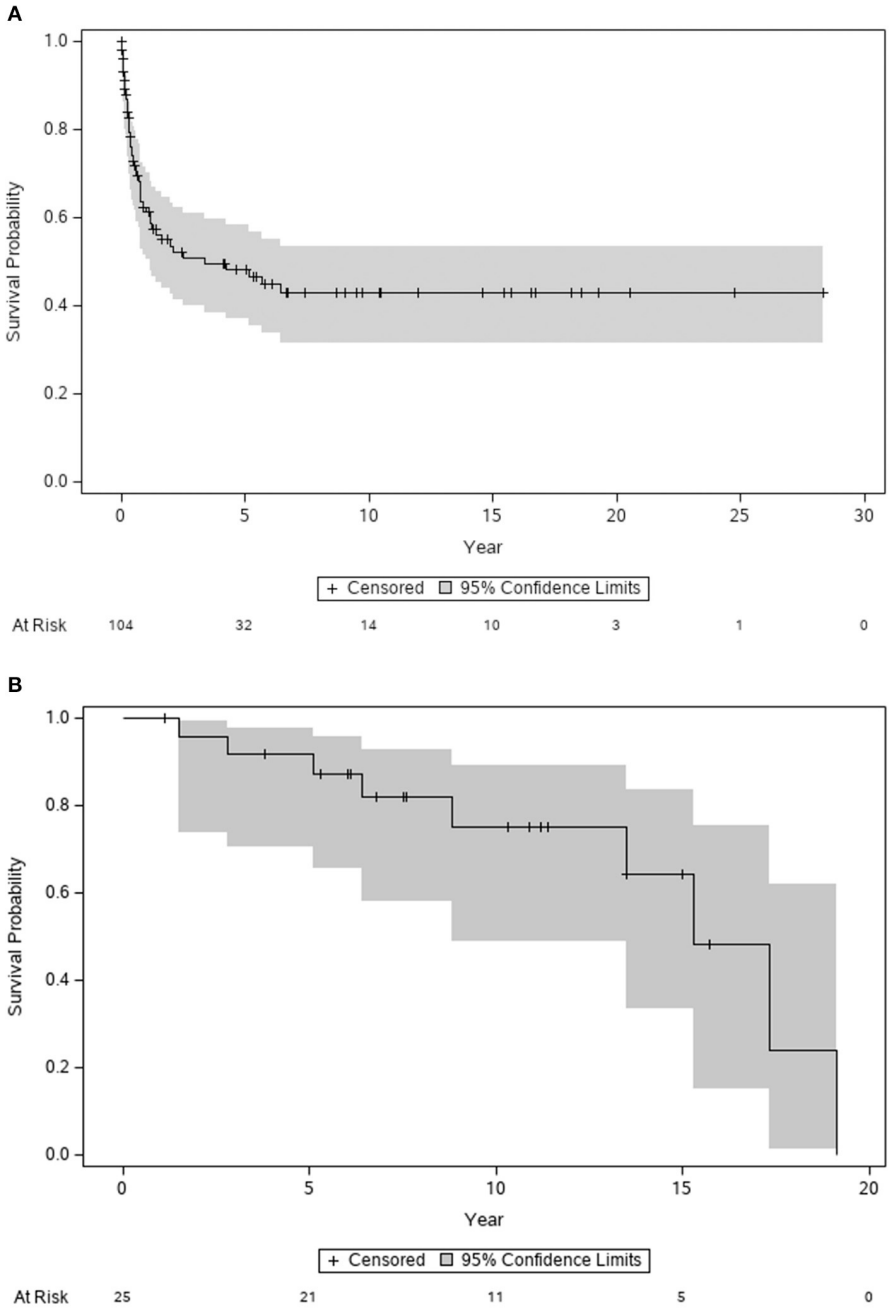
Transplant-free survival **(A)** and post-transplant survival **(B)** for patients with primary dilated cardiomyopathy. Gray shaded, 95% confidence intervals.

The 1-, 5-, and 10-year transplant-free survival rates were 61.1, 48.0, and 42.8%, respectively (Figure 1A). In patients who received transplantation, the 1-, 5-, and 10-year survival rates after the transplant were 100, 91.7, and 75.0%, respectively (Figure 1B). The transplant-free survival and survival after transplantation were comparable between those diagnosed in the early and late eras.

### Recovery

**Figure 2:**
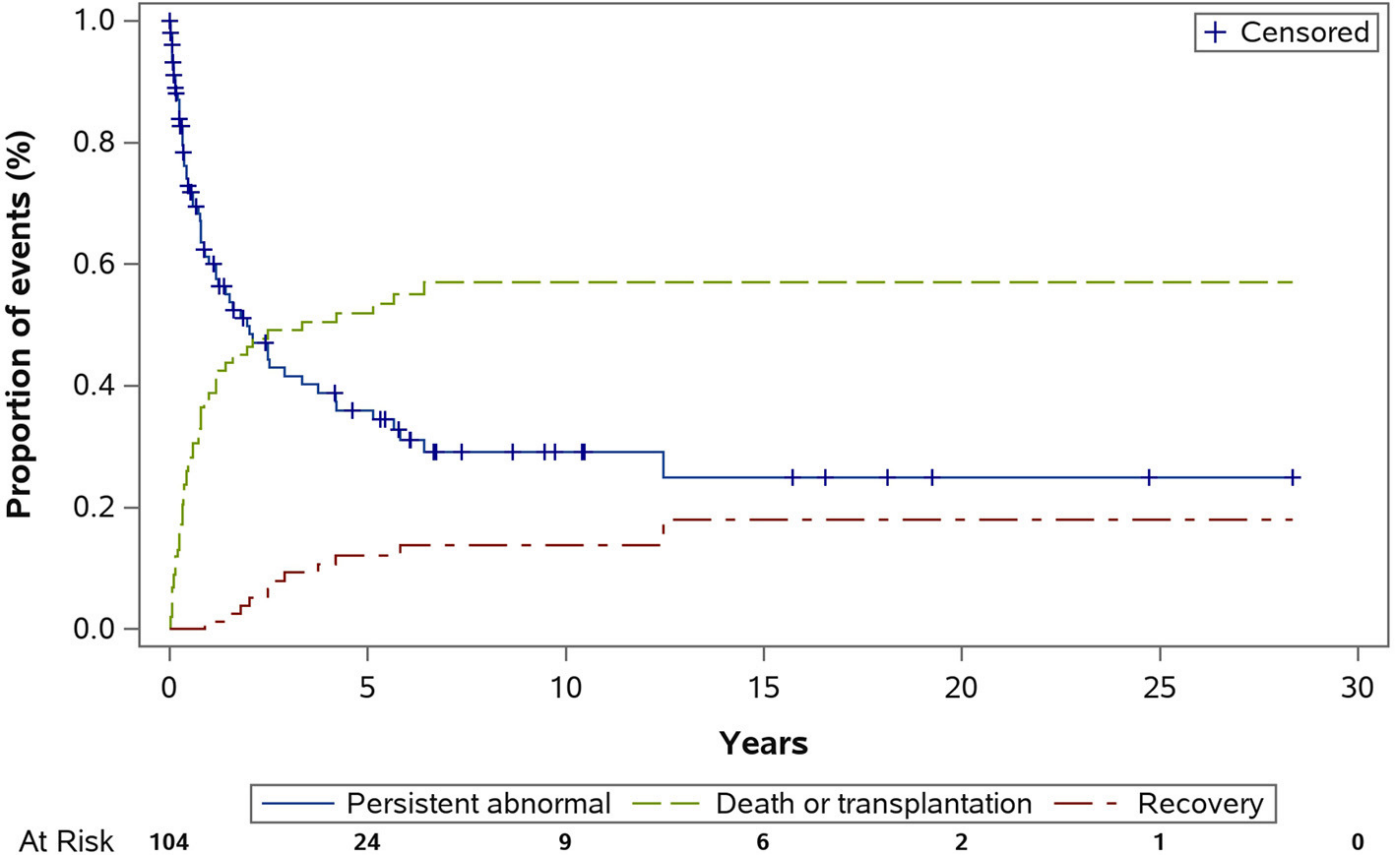
Cumulative incidence of left ventricular normalization (recovery) in patients with primary dilated cardiomyopathy (Competing risk factors, death or transplant).

In total, the LV function of 11 patients (10.6%) recovered after a median follow-up of 2.5 years (IQR = 1.65–5.00 years). The cumulative incidence rates of LV function recovery in the presence of competing risk factors, including death or transplantation, are depicted in Figure 2. The cumulative incidence rate of recovery at 1, 3, and 5 years after the diagnosis was 1.2, 9.3, and 12.1%, respectively. Patients with LV function recovery during follow-up were significantly younger at the initial diagnosis than those without recovery (2.1 ± 2.5 vs. 4.8 ± 5.8 years, *P* < 0.001). No other significant differences were observed in clinical characteristics or electrocardiogram or echocardiographic parameters. Additionally, two patients had LV function recovery after intervention. One underwent pulmonary artery banding at the age of 4 months, and recovery was documented 5 months after the surgery (11). The other underwent cardiac resynchronization therapy at the age of 3 years, and recovery was documented 6 months later (12).

### Impacts of Age on Clinical Features and Outcomes

To investigate the potential influences of age at diagnosis on primary and secondary outcomes, receiver operating characteristics curve analysis was performed. The cutoff age of 3 years had the highest power for predicting death or transplantation, with the area under the curve being.69 (95% CI:0.58–0.79, *P* = 0.001). Regarding age in relation to LV function recovery, the area under the receiver operating characteristics curve was not significant.

**Figure 3:**
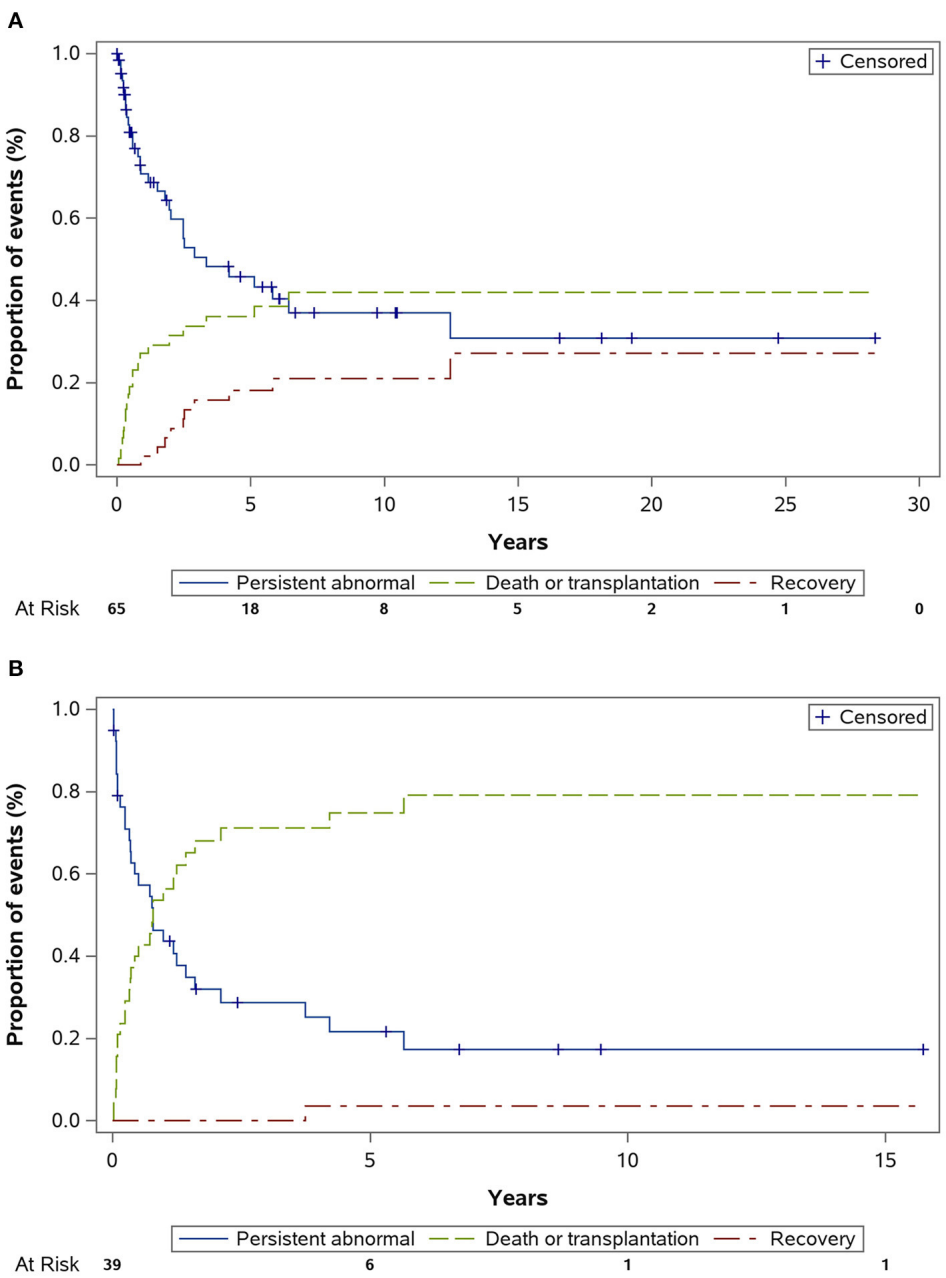
Cumulative incidence of left ventricular normalization (recovery) by age. (Competing risk factors, death or transplant). Estimated cumulative incidence rate of recovery was higher in those diagnosed before age 3 years **(A)** than those diagnosed after age 3 years **(B)**, *P* = 0.028.

Significant differences in clinical features were observed between patients <3 (*n* = 65) and ≥3 (*n* = 39) years (Table 2). The number of female patients was higher in the younger group (61.5%), whereas the male:female ratio was 3.3 in the older group. The initial presentation of clinical symptoms differed significantly between these two groups. Furthermore, patients in the younger group had larger LV EDD and LV ESD *Z* scores at diagnosis. The application of either LVAD or ECMO was more prevalent in the older aged group. Among various classes of medications, we found that digoxin was prescribed more frequently in younger aged group, while there was no difference in the prescription frequency among other medications. Considering clinical outcomes at the end of the follow-up, patients aged >3 years at diagnosis were at higher risk of either death or transplantation than those aged <3 years of age (71.8 vs. 32.3%, *P* < 0.001). In addition, patients in the older group had a lower chance of LV function recovery (15 vs. 3%, *P* = 0.028). The cumulative incidences of LV function recovery based on age at diagnosis are shown in Figure 3. Younger patients were less likely to be included in the transplantation waitlist, and they had a significantly lower chance of receiving heart transplantation once listed (5/23 vs. 20/25, *P* < 0.001); thus, the waitlist mortality rate was significantly higher in the younger group (10/23 vs. 3/25, *P* = 0.022) compared with the patients aged >3 years.

**Table 2:** Comparisons between patients with a diagnostic age <3 years and ≥3 years.

	**<3 years** **(*n* = 65)**	**≥3 years** **(*n* = 39)**	***p*-value**
Male, *n* (%)	25 (38.5)	30 (76.9)	**<0.001**
**Main presenting symptoms (*****n*** **= 72)**			**0.003**
Dyspnea, *n* (%)	21 (32.3)	16 (41.0)	
Poor activity/appetite, *n* (%)	20 (30.8)	2 (5.1)	
Nausea, vomiting, or abdominal pain, *n* (%)	3 (4.6)	7 (17.9)	
Failure to thrive, *n* (%)	3 (4.6)	0	
QRS prolongation, *n* (%)	12 (18.5)	7 (17.9)	0.95
**Echocardiography parameters at diagnosis**			
Severe MR, *n* (%)	13 (20.0)	9 (23.1)	0.71
LV EDD, *Z* scores	8.0 ± 3.7	5.6 ± 2.2	**<0.001**
LV ESD, *Z* scores	10.7 ± 4.3	9.2 ± 3	**0.042**
LV EF	30 ± 9.2	28.8 ± 9.6	0.52
Clinical outcomes^*^			
ECMO, *n* (%)	7 (10.8)	10 (25.6)	**0.047**
LVAD, *n* (%)	5 (7.7)	11 (28.2)	**0.005**
Recovery, *n* (%)	10 (15.4)	1 (2.6)	**0.028**
On waitlist, *n* (%)	23 (35.4)	25 (64.1)	**0.004**
Transplantation, *n* (%)	5 (7.7)	20 (51.3)	**<0.001**
Waitlist mortality, *n* (%)	10 (43.5)	3 (12.0)	**0.022**
Death or transplantation, *n* (%)	21 (32.3)	28 (71.8)	**<0.001**

*ECMO, extracorporeal membrane oxygenation; MR, mitral regurgitation; LVAD, left ventricular assist device; LVEDD, left ventricular end-diastolic dimension; LVEF, left ventricular ejection fraction; LVESD, left ventricular end-systolic dimension*.

* *At the latest follow-up visit*.

*P < 0.05 were shown in bold*.

### Prognosticator for Clinical Outcomes

**Figure 4:**
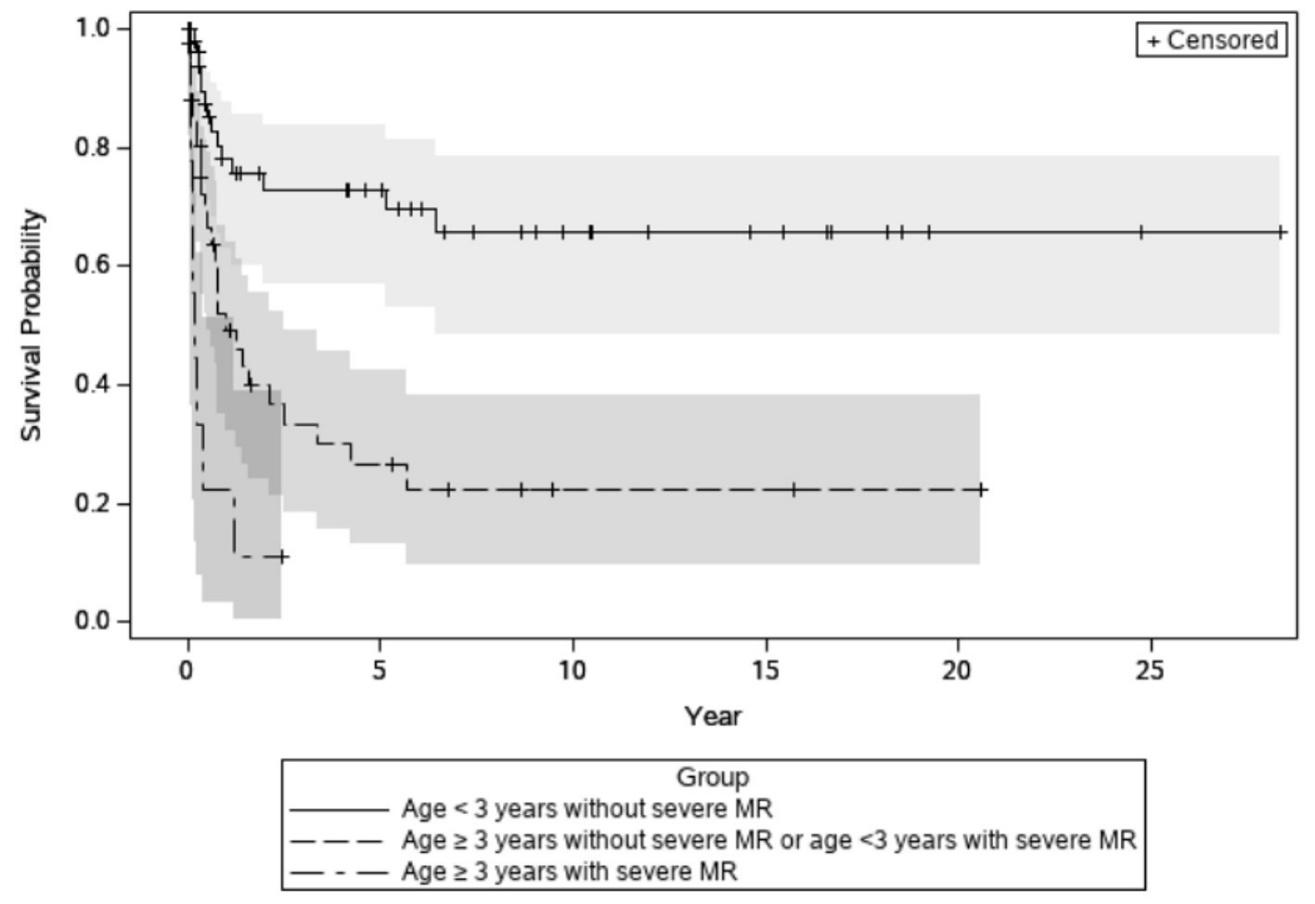
Transplant-free survival stratified by risk factors. Transplant-free survival of those without risk factors, namely severe mitral regurgitation and age at diagnosis ≥3 years, was significantly higher than those with one or two risk factors. Both *P* < 0.001. There was no significant survival difference between those with one risk factor and those with two risk factors, *P* = 1.00. Gray shaded, 95% confidence intervals.

In the univariate analysis, age at diagnosis ≥ 3 years, male sex, severe MR, and lower LV EF at the initial diagnosis were significant predictors of mortality or transplantation (Table 3). In the multivariate Cox regression analysis, only age ≥ 3 years (adjusted hazard ratio = 2.93, 95% CI = 1.66–5.2, *P* < 0.001) and severe MR at diagnosis (adjusted hazard ratio = 3.31, 95% CI = 1.77–6.2, *P* < 0.001) were independent predictors of death or transplantation. Transplantation-free survival curves, stratified based on diagnostic age and MR severity, are displayed in Figure 4.

**Table 3:** Hazard ratios from univariate and multivariate Cox regression model of risk factors for death or transplantation.

	**Univariate analysis**			**Multivariate analysis**		
	**HR**	**95% CI**	***P-*value**	**HR**	**95% CI**	***P-*value**
Age ≥ 3 yr	2.93	1.66–5.18	**<0.001**	2.93	1.66–5.2	**<0.001**
Male	1.98	1.09–3.61	**0.025**	1.45	0.72–2.92	0.29
QRS prolongation			0.57			
Severe MR	3.31	1.78–6.16	**<0.001**	3.31	1.77–6.2	**<0.001**
LV EDD, *Z* scores			0.68			
LV ESD, *Z* scores			0.28			
LV EF	0.97	0.94–1.00	**0.048**	0.98	0.95–1.01	0.13

*CI, confidence interval; HR, hazard ratio; MR, mitral regurgitation; LV EDD, left ventricular end-diastolic dimension; LV EF, left ventricular ejection fraction; LV ESD, left ventricular end-systolic dimension. The bold values indicates statistically significant p values*.

## Discussion

We report long-term outcomes of pediatric primary DCM in an Asian population. Large-scale studies on Asian populations have been few, and the organ donation rate in Asia is low. As the largest pediatric cardiac center and heart transplantation center in Taiwan, we compared long-term survival and survival after transplantation in the Asian population with those in published reports from western countries (3, 13). In addition, we found that age at diagnosis had significant effects on both clinical outcomes and access to heart transplantation in this Asian population. The results from our study may aid risk stratification and clinical decision making regarding this severe disease.

Reports from western countries have revealed that the transplant-free survival rates among pediatric patients with primary DCM at 1 and 5 years are 61–82% and 47–72%, respectively (Table 4) (3, 13). The present study discovered comparable survival rates in an Asian cohort, with the 1- and 5-year survival being 61 and 48%, respectively. Although improved survival in the recent era has been noted in a multicenter registry in North America (5). We did not find a significant difference in survival between early and late diagnostic eras in our study cohort. Beta-blockers were used more frequently in our cohort from the recent era. However, a randomized, double-blind, placebo-controlled trial (17) showed that beta-blockers do not significantly improve clinical heart failure outcomes in children and adolescents with symptomatic heart failure. Furthermore, a retrospective review revealed that the combination of angiotensin-converting enzyme inhibitor and beta-blocker therapy was not associated with significant improvement of transplantation-free survival compared with conventional digoxin-based therapy in a pediatric population (13). The outcome of pediatric DCM may be likely based on underlying patient characteristics and disease severity rather than the choice of medical therapy.

**Table 4:** Comparisons of transplant-free survival rates, recovery rates, and waitlist mortality rates among western countries and the present Asian cohort.

	**Transplant-free survival**		**Recovery**	**Waitlist mortality**
	**1-year**	**5-year**		
United States (3, 14, 15)	61%	47%	22%	11%
Canada (13)	69.7%	52%	NM	NM
Australia (4)	74%	65%	69%	NM
Netherland (16)	82%	72%	53%	NM
Taiwan	61%	48%	20%	27%

*NM, not mentioned*.

We demonstrated that age at diagnosis is a crucial prognosticator of clinical outcomes in an Asian cohort of pediatric primary DCM. At a younger age, although the likelihood of receiving heart transplantation is low and waitlist mortality is high, younger age at diagnosis (<3 years) was independently related to lower risk of mortality and transplantation in our cohort. Lower incidence of either ECMO or LVAD implantation in younger aged patients might also reflect more favorable clinical outcomes in this patient subgroup. This is concordant with several reports from western countries (3, 18–21). In addition, we demonstrated that younger age at diagnosis may be related to higher probability of LV function recovery. Moreover, this finding is similar to that reported in the US Pediatric Cardiomyopathy Registry (14). The exact explanation for this observation is unclear. In our cohort, female patients were significantly more predominant than male patients among the younger patients, whereas the opposite was the case in the older group. Such reversal in sex predominance in different age groups has not been reported before. In pediatric DCM, male sex has been reported to be associated with higher disease incidence (2, 3) and increased risk of disease progression (22). Additionally, younger patients may be more likely to have underlying inborn metabolism anomalies or malformation syndromes, which are generally associated with better survival (3). Further studies incorporating more comprehensive data derived from genetics-based diagnostic tools are required to explore the mechanistic explanation of age's effects on the outcomes of pediatric DCM.

Echocardiography plays an essential role in outcome prediction for pediatric DCM. We found that of the various echocardiographic parameters, severe MR was independently related to death or transplantation. This is compatible with the finding of a systematic review conducted by Alvarez et al. (18). Other reports have shown that poor LV EF can contribute toward identifying patients at risk of poor outcomes (19). In our analysis, a low LV EF at initial diagnosis was predictive of death or transplantation in the univariate analysis. However, LV EF became a significant predictor in the multivariate regression analysis only when MR severity was excluded (data not shown). In cases of severe MR, LV EF may overestimate LV systolic function because of altered loading conditions. This phenomenon may explain why LV EF lost its predictive role when severe MR was considered. Surgery for severe MR was performed in a few patients in our study. Reports have demonstrated that mitral valve repair is safe and can decrease the cardiac dimension and improve symptoms in children with DCM (23, 24). Although MR is crucial in predicting outcomes, whether it is a modifiable factor in relation to clinical outcomes remains uncertain. Further studies are needed to ascertain the benefits of surgery.

Racial and ethnic differences are considered crucial factors contributing to different clinical phenotypes and prognoses in pediatric DCM (2, 3, 25). Although outcome predictors are similar between our patient cohorts and previous Western patient populations, some differences were indeed observed, such as a distinct difference in sex predominance between younger and older groups and a lower rate of LV function recovery (Table 4). In addition to potential biological factors underlying racial differences, cultural factors linked to racial differences may influence pediatric DCM outcomes. The transplantation rate (52.1%) in our cohort was much lower than that in western countries (75%), and the waitlist mortality rate was exceptionally high in our study (27%), as opposed to 11% in another report (Table 4) (15). This is largely attributed to there being few heart donors in Taiwan, particularly infants and young children. Several major barriers to organ donation have been noted in Asian populations (26–28). East Asians traditionally desire an intact body at death to comply with the concept of filial piety within Confucianism (26). Furthermore, religions such as Buddhism and Taoism play crucial roles in decision-making regarding organ donation (29). A survey in Taiwan revealed that many donor families experienced negative psychocognitive bereavement because of their decision to donate (30). Because the survival after transplantation in our study was comparable with that in contemporary registry data (15), we believe that the overall survival of pediatric DCM in this Asian population can be improved through public education and promotion of organ donation.

### Limitations of This Study

This was a retrospective study investigating participants from a single institution. Although our institute is the single largest pediatric cardiac center in Taiwan, referral bias could not be completely avoided. For example, there were nine patients (8.7%) who were referred to our hospital specifically for heart transplant evaluation. Besides, lack of routine genetic testing throughout the study period is another limitation of this study. Although we excluded patients with a clinical history of acute myocarditis, histology evaluation was usually not performed in our study subjects. Although our single-institute database enabled us to collect detailed data on the use of heart failure medications, information related to the dosage and treatment duration was insufficient for analyzing their association with clinical outcomes. Lastly, we did not collect data on right ventricular function, which were not consistently assessed in our previous evaluation.

## Conclusions

In this Asian pediatric DCM cohort, the transplant-free survival and survival after heart transplantation were comparable with those in other reports involving Western populations. Approximately half of the patients were on the transplant waitlist, but only half of them eventually received transplantation. Although younger patients had a lower chance of receiving heart transplantation and higher waitlist mortality, lower age at diagnosis was associated with better survival and higher likelihood of LV function recovery. Distinct difference in the predominant sex between the younger and older patient groups and lower rate of LV function recovery were unique features of this Asian pediatric DCM cohort. The information provided by our study can assist risk stratification and clinical decision making in pediatric primary DCM.

## Data Availability Statement

The original contributions presented in the study are included in the article/supplementary material, further inquiries can be directed to the corresponding author/s.

## Ethics Statement

The studies involving human participants were reviewed and approved by Research Ethics Committee B, National Taiwan University Hospital. Written informed consent from the participants' legal guardian/next of kin was not required to participate in this study in accordance with the national legislation and the institutional requirements.

## Author Contributions

P-YW: study design, data acquisition, analysis, interpretation, writing of manuscript, and final approval of the manuscript. W-CT: analysis, interpretation, writing of manuscript, and final approval of the manuscript. C-MF: analysis, interpretation, and final approval of the manuscript. J-KW, M-HW, Y-SC, N-KC, S-SW, M-TL, C-WL, and S-NC: patient enrollment and follow-up, critical appraisal of the data, and final approval of the manuscript. C-AC: responsible for all aspects of the project. All authors contributed to the article and approved the submitted version.

## Funding

This study was supported by grants from Taiwan Ministry of Science and Technology (MOST 108-2314-B-002-005) and National Taiwan University Hospital (the bridging project, 110-17).

## Conflict of Interest

The authors declare that the research was conducted in the absence of any commercial or financial relationships that could be construed as a potential conflict of interest.

## Publisher's Note

All claims expressed in this article are solely those of the authors and do not necessarily represent those of their affiliated organizations, or those of the publisher, the editors and the reviewers. Any product that may be evaluated in this article, or claim that may be made by its manufacturer, is not guaranteed or endorsed by the publisher.
